# Efficacy and safety of secukinumab for the treatment of severe ABCA12 deficiency‐related ichthyosis in a child

**DOI:** 10.1002/ski2.25

**Published:** 2021-05-03

**Authors:** J. Yogarajah, C. Gouveia, J. Iype, S. Häfliger, A. Schaller, J.M. Nuoffer, M. Fux, M. Gautschi

**Affiliations:** ^1^ Division of Paediatric Endocrinology, Diabetology and Metabolism Department of Paediatrics University Hospital Bern Inselspital Bern Switzerland; ^2^ Department of Dermatology University Hospital Bern Inselspital Bern Switzerland; ^3^ University Institute of Clinical Chemistry University Hospital Bern Inselspital Bern Switzerland; ^4^ Department of Human Genetics University Hospital Bern Inselspital Bern Switzerland

## Abstract

**Background:**

Patients with severe autosomal recessive congenital ichthyosis (ARCI) show a T helper 17/interleukin 17 (Th17/IL17) skewing in their skin and serum, resembling the inflammatory profile of psoriatic patients. Secukinumab, an IL‐17A inhibitor, has shown clinical efficacy in patients with moderate‐to‐severe plaque psoriasis.

**Aims:**

To test the clinical efficacy and safety of secukinumab in a paediatric patient with ATP‐binding cassette subfamily A member 12 deficiency‐related severe erythrodermic ARCI.

**Materials & Methods:**

6‐months therapeutic trial. During the first 4‐weeks induction period, the patient received weekly subcutaneous injections of 150 mg secukinumab (five injections in total). During the following 20‐weeks maintenance period, the patient was given a subcutaneous injection of 150 mg secukinumab every 4 weeks.

**Result & Discussion:**

After the 6‐months therapy period, there was a 48% reduction from the baseline Ichthyosis‐Area‐Severity‐Index (‐Erythema/‐Scaling) score. The treatment was well tolerated. Moreover, cytokine analysis revealed a reduction of keratinocyte‐derived proinflammatory cytokines and an abrogation of Th17‐skewing during therapy.

**Conclusion:**

Further studies are needed to evaluate the effects of the use of IL‐17A inhibition in ARCI patients.

## Graphical abstract

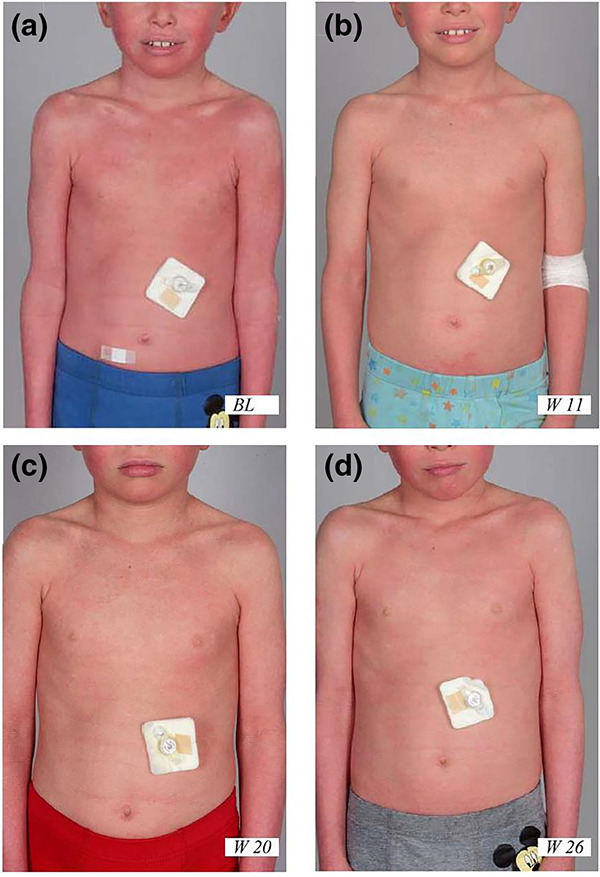

We present a pediatric patient with ABCA12 deficiency‐related severe erythrodermic Ichthyosis who responded well to a six months individual trial with secukinumab. The treament was well tolerated. Clinical efficacy was accompanied by a consistent shift in the cytokine profile in the patient serum.

1

**What is already known about this topic?**

- In patients with ichthyosis, a T helper 17/interleukin 17 (IL‐17) skewing in skin and serum has been demonstrated, resembling the expression profile of psoriatic patients.
- IL‐17A inhibitor is an approved biologic treatment for patients with moderate to severe plaque psoriasis, not yet for patients with ichthyosis.

**What does this study add?**

- Secukinumab was effective and well tolerated in the treatment of a paediatric patient with ATP‐binding cassette subfamily A member 12 deficiency‐related severe erythrodermic ichthyosis.
- Clinical efficacy was accompanied by a consistent shift in the cytokine profile in the patient serum.
- The use of secukinumab in ichthyosis represents a promising therapeutic approach.

## 2 INTRODUCTION

Autosomal recessive congenital ichthyoses (ARCI) include a group of heterogeneous keratinisation disorders. The clinical manifestations range from relatively mild lamellar ichthyosis and erythrodermic ARCI to the most severe, harlequin ichthyosis.^1^

In the granular layer keratinocytes, the ATP‐binding cassette subfamily A member 12 (ABCA12) enables the transport of lipids into the lamellar granules whose content is released into the interstitial lipid domain between the terminally differentiated keratinocytes.^2^ The intercellular lipid layers (ILLs) in the stratum corneum are essential to maintain the epidermal skin barrier function.^3^ Complete ABCA12 deficiency causes harlequin ichthyosis, whereas variants with residual activity lead to ARCI manifestations,^4^ due to defective ILL formation, keratinocyte differentiation and skin desquamation, causing epidermal skin barrier disruption and hyperkeratosis.^3^

Keratinocytes are the major target of the proinflammatory interleukin 17A (IL‐17A),^5^ which is primarily produced by T helper cells type 17 (Th17)^6^ (Figure S1). IL‐17A has been recognised as a key cytokine that drives inflammatory pathways in the pathogenesis of psoriasis.^5^ Secukinumab, an IL‐17A inhibitor, has been approved for the treatment of patients with moderate to severe plaque psoriasis.^7^ In patients with ichthyosis, a Th17/IL‐17 skewing in skin and serum has been demonstrated, resembling the expression profile of psoriatic patients.^8^, ^9^ Hence, the therapeutic approach for psoriasis may also be promising in patients with ichthyosis.^9^

Here we present, to our knowledge, the first paediatric patient with ABCA12 deficiency‐related erythrodermic ichthyosis who showed relevant improvement under treatment with secukinumab. The patient's severe skin condition with generalised erythema and scaling required extensive and time‐consuming, yet insufficient therapy. Given the patient's considerably impaired quality of life, we performed an individual off‐label 6‐months therapeutic trial with secukinumab. The treatment was continued afterwards and is still maintained.

## 3 MATERIALS AND METHODS

Written informed consent for both the off‐label treatment and publication of the case, including the pictures, was obtained from the patient family. All procedures were performed in accordance with local institutional review board requirements. Approval by the responsible ethics committee was waived.

This individual off‐label 6‐months therapeutic trial involved an 8‐year‐male patient with the clinical diagnosis of erythrodermic ARCI (see Case History in Supporting Information Material). The skin biopsy showed dermatitis with psoriasis‐like characteristics. The symptomatic therapy with different emollients and mild keratolytic was continued during the trial with no change apart from frequency and duration of topical skin care, which were adapted to need.

During the first 5‐weeks induction period, the patient (body weight 21 kg) received weekly subcutaneous injection of 150 mg secukinumab (Cosentyx^®^; Novartis Pharma AG), followed by a 20‐weeks maintenance period, with injections every 4 weeks, according to the manufacturer's recommendations.

Visits to the clinic took place on Weeks 0 (baseline), 2, 4, 8, 11, 16, 20 and 26. The clinical efficacy of secukinumab was assessed by standardised pictures of the skin, as well as a battery of clinical scorings. The safety of the drug was evaluated by general physical examination and diary of adverse events, including intercurrent illnesses, infections and other known side effects of secukinumab. Safety laboratory analyses were performed.

Measurement of cytokines in serum: Serum samples were taken from the patient before and at 1, 3 and 6 months of therapy. *For further information, see eMethods in the Supporting Information Materials*.

## 4 RESULTS

The treatment was generally well tolerated with no side effects or new symptoms during the whole 6‐months treatment period. Safety laboratory analyses showed no adverse findings.

**FIGURE 1:**
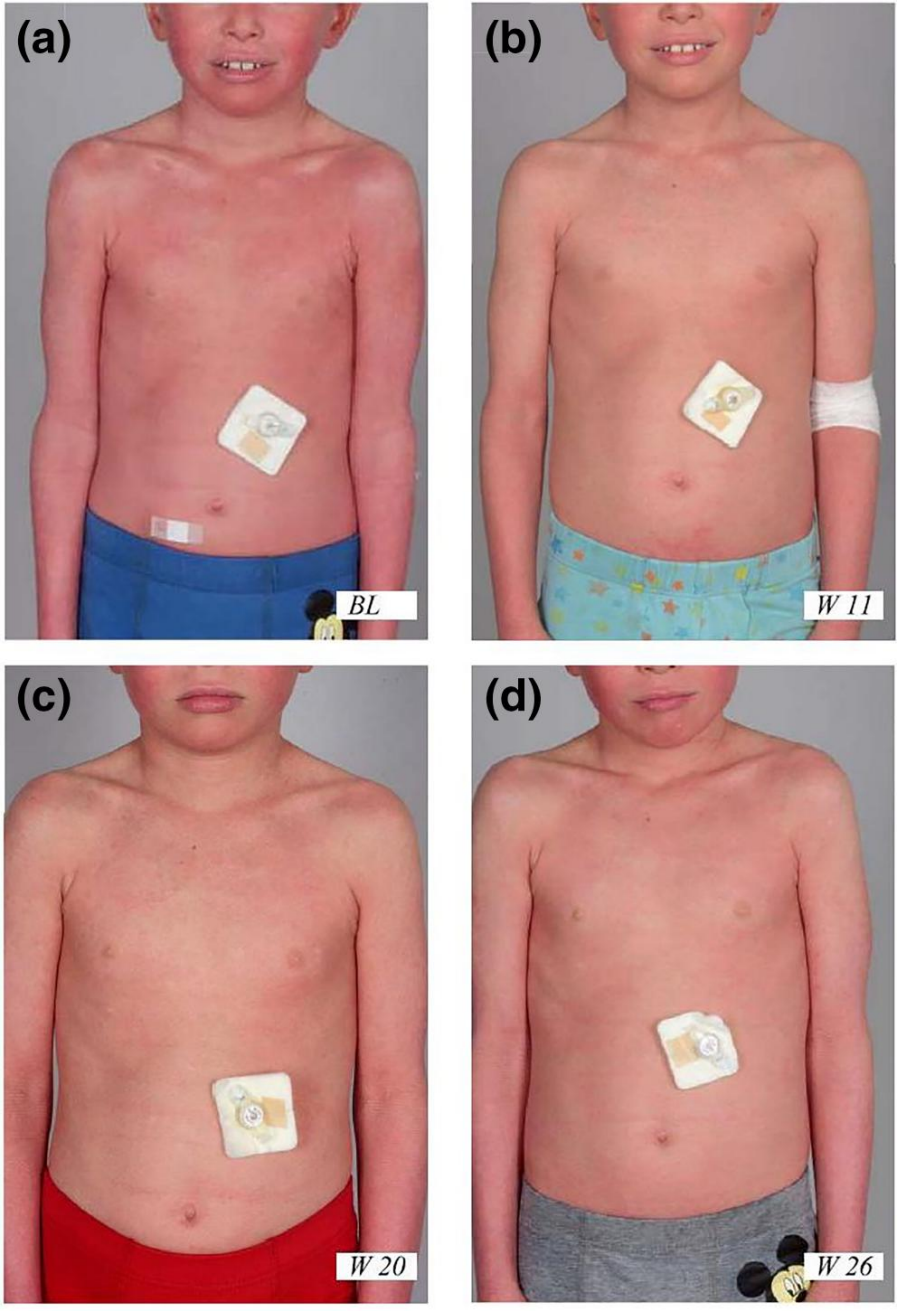
Clinical follow up. Pictures taken at baseline visit (a), and after 11 (b), 20 (c) and 26 (d) weeks of treatment, respectively, show its clear and persistent efficacy with albeit varying residual disease activity. The gastrostomy button on the left abdomen was inserted for the treatment of underfeeding during early childhood (with kind permission of the patient and his parents)

Clinical follow‐up is summarised in Table 1. Baseline scores of Investigator's Global Assessment and Clinical Global Impression (‐Severity) (CGI‐S) reflected the patient's severe condition (Table 1). Under treatment, scaling and erythema declined. Only 4 weeks after the start of therapy, there was a 38% reduction of the baseline Ichthyosis‐Area‐Severity‐Index score (Table 1). At 8 weeks follow‐up visit, the skin regained a rough, scaly and erythematous appearance (Table 1), with recurrence of hyperkeratosis, but of a decreased area in all body regions. At 11 and 16 weeks, the skin condition showed again minimal improvement (CGI‐I 3), with mild palmoplantar hyperkeratosis. Both erythema and scaling decreased, while the facial area remained erythematous (Figure 1). At 20 weeks follow‐up visit, the skin remained slightly scaly, but altogether the skin condition improved much (CGI‐I 2) and became more stable.

**TABLE 1:** Clinical follow up using a battery of scoring systems

		Induction Period						Maintenance Period									
Month						1		2		3		4		5		6	
Week		0	1	2	3	4		8	11	12		16		20		24	26
Injection		S	S	S	S	S		S		S		S		S		S	
Follow‐Up Visits		V		V		V		V	V			V		V			V
Scoring	[range]																
IGA	[0,5]	4				3			3			3		3			4
CGI Severity	[1,7]	5				4			4			4		4			3
CGI Improvement	[1,7]					2			3			3		2			2
IASI‐E/S	[0,48]	28.2				17.4		19.2	12.4			10.1		12.8			13.5
‐ Erythema	[0,24]	18.0				12.0		12.0	8.4			7.4		6.4			5.5
‐ Scaling	[0,24]	10.2				5.4		7.2	4.0			2.7		6.4			8
Itch‐Man Scale	[0,4]	1				2		2	2			2		3			2
Wong‐Baker Faces	[0,10]	2				2		4	2			2		4			4
CDLQI	[0,30]	6				12		10	7			7		9			4
TSQM‐9	[9,59]					43			44			41		42			45

*Note*: Further follow‐up controls were carried out in the Weeks 2, 4, 8, 11, 16, 20 and 26.

Abbreviations: CDLQI, Children's Dermatology Life Quality Index; CGI (‐S/‐I), clinical global impression (‐severity/‐improvement); IASI (‐E/‐S), Ichthyosis‐Area‐Severity‐Index (‐Erythema/‐Scaling); IGA, Investigator's Global Assessment; QOL, quality of life; S, secukinumab injection; TSQM‐9, abbreviated 9‐item Treatment Satisfaction Questionnaire for Medication; V, visit.

Under secukinumab treatment, the required time for the basic therapy was shortened by 20 min/day. Occasionally and towards the end of the 6‐months therapy period, daily bathing could be skipped and the extent of topical treatment could be reduced. The maculopapular rash was milder and observed only intermittently for a few days. With the addition of an oral antihistaminic treatment during the second month of secukinumab treatment, the itching became tolerable. Moreover, the regular ear cleanings had become more manageable, as the ear canals were less moist and inflamed.

**FIGURE 2:**
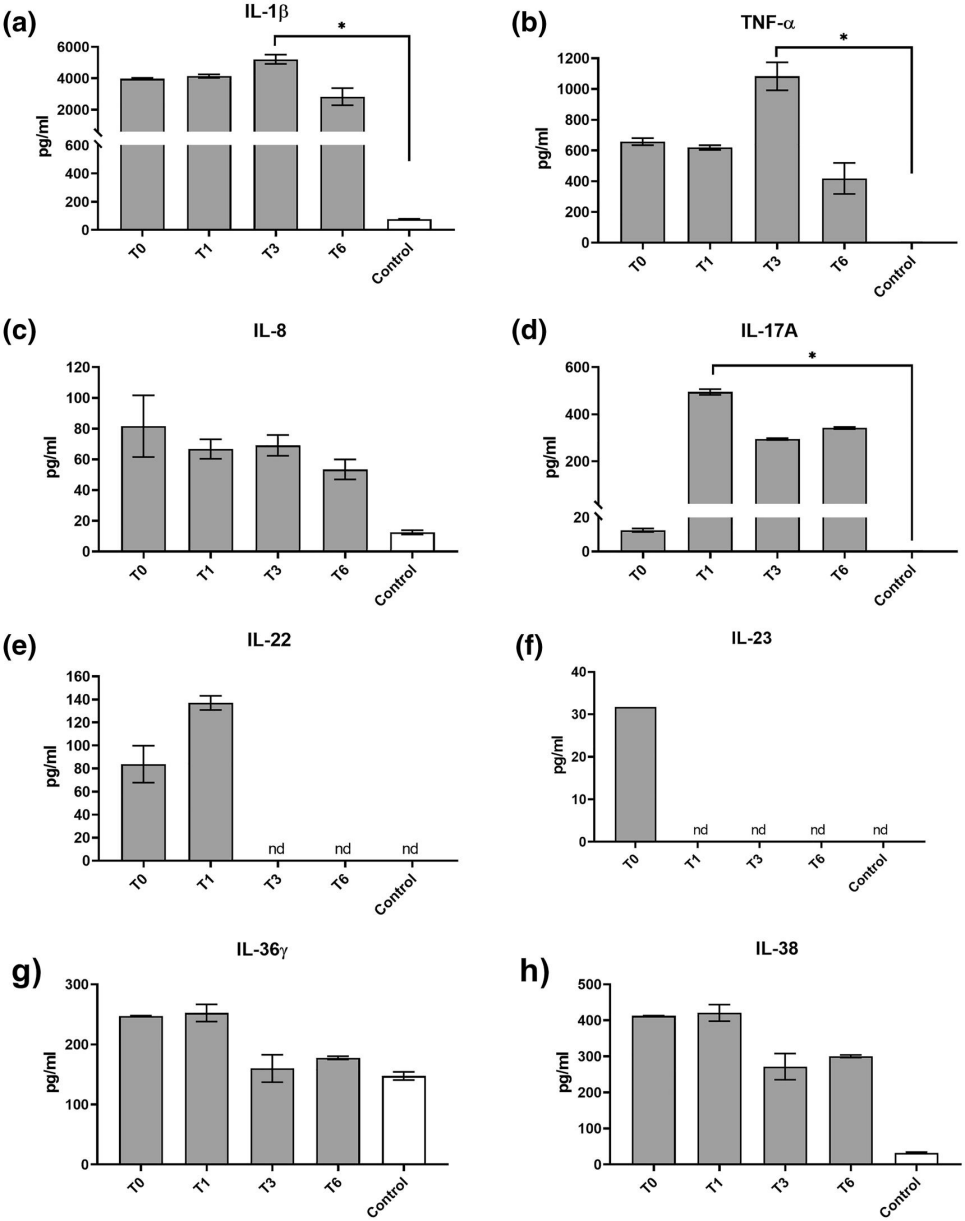
Serum cytokine profiles of the patient before and under anti‐IL‐17 antibody therapy. Quantification of serum cytokines, IL‐1*β* (a), TNF‐*α* (b), IL‐8 (c), IL‐17A (d), IL‐22 (e), IL‐23 (f), IL‐36*γ* (g), and IL‐38 (h) using ELISA. Data shown are of patient's samples (shaded bars) collected before (T0) and at 1, 3 and 6 months (T1, T1, T6) of anti‐IL‐17 antibody therapy. Controls are the mean from six age‐matched control subjects with presumably normal cytokine profile (for details see eMethods in Supporting Information Materials). All data are shown as mean ± SEM of replicates/data sets. ELISA, enzyme‐linked immunosorbent assay; IL, interleukin; nd, not detectable; TNF‐*α*, tumour necrosis factor *α*. *p < 0.05 by unpaired nonparametric Kruskal–Wallis test with Dunnett's multiple group comparison

Results of the cytokine analysis in patient's sera are shown in Figure 2. It revealed a milieu strongly enriched in pro‐inflammatory Th1 cytokines IL‐1*β*, tumour necrosis factor *α* (TNF‐*α*) and IL‐8 before treatment. Moreover, a Th17 skewing was observed as evidenced by the presence of the Th17 differentiating cytokine IL‐23 and the Th17 cell‐derived cytokines IL‐17A and IL‐22. IL‐36*γ*, a putative biomarker of psoriasis, and IL‐38, an antagonist of IL‐36*γ*, were also detectable in patient's serum at time point 0. During treatment, we observed a significant reduction of IL‐22 and IL‐23 in patient's sera. In contrast, the levels of all other tested cytokines remained high during the administration of secukinumab. *For further information*, see *Case History in the Supporting Information Material*.

## 5 DISCUSSION

In children with congenital non‐syndromic ichthyosis, secukinumab has not been used so far, and no clinical trials have assessed its safety and efficacy in children in general. Two paediatric case reports showed the successful use of secukinumab in IL‐36 antagonist deficiency with generalised skin eruptions and in psoriatic arthritis.^10^, ^11^ There is an ongoing randomised, double‐blind phase II clinical trial (NCT03041038) on the efficacy and safety of a 16‐weeks use of secukinumab in adult patients with ichthyosis (https://clinicaltrials.gov/ct2/show/NCT03041038, last accessed on 18 August 2020). Meanwhile, Hernandez‐Martin et al.^12^ have very recently published their clinical experience with one infant with a severe skin dermatitis, multiple allergies and metabolic wasting‐like syndrome who showed clinical improvement under secukinumab.

This individual trial shows the efficacy and safety of secukinumab for the treatment of ABCA12 deficiency‐related ichthyosis in a child. Secukinumab has been reported to be fast and effective in the reduction of psoriasis symptoms.^7^ During the induction period, this prompt efficacy was observed in our patient; erythema and scaling, showed striking improvement. With the transition to monthly injections, the skin condition improved again and stabilised after an initial transient deterioration. Our patient had no increased or opportunistic infections nor any other occurrence suggestive of an adverse drug reaction.

Patients with ichthyosis are reported to have a higher concentration of IL‐17A.^8^, ^9^ Accompanying this individual therapeutic trial, we have performed a cytokine profiling**.** Interestingly, the levels of the proinflammatory Th1 cytokines IL‐1*β* and TNF‐*α* were 40‐ and 30‐fold increased, respectively, compared to studies of patients with other dermatological conditions, including paediatric ichthyosis^13^ and adult psoriasis.^14^ Moreover, these Th1 cytokine levels remained high during treatment underscoring the prolonged systemic Th1 inflammation in our patient. Moreover, a Th17 skewing was observed as evidenced by the presence of the Th17 differentiating cytokine IL‐23 and the Th17 cell‐derived cytokines IL‐17A and IL‐22. IL‐36*γ*, which has been described as a biomarker of psoriasis^15^ and IL‐38, an antagonist of IL‐36*γ*, were also detectable in patient's serum at time point 0. Consistently, recently published studies report high expression of IL‐36*γ* in skin samples obtained from ichthyosis^16^ and psoriasis patients.^17^ Moreover, treatment with secukinumab resulted in reduced systemic and local IL‐36*γ* expression accompanied by an upregulation of IL‐38.^17^ Also, the study of Enjalbert et al.^16^ describes an opposing expression of IL‐36*γ* and its antagonist IL‐37, whereby the former is expressed at high and the later at low levels in patient's skin. Interestingly, IL‐36*γ* and IL38 levels declined during secukinumab therapy in our patient indicating that the agonist‐antagonist axis of IL‐36*γ* and IL‐38 seems not to play a role here.

We detected increased levels of IL‐17A following secukinumab therapy. We assume that these detected higher levels of IL‐17A were due to complex formation between IL‐17A and secukinumab. Secukinumab‐IL‐17A complexes are less efficiently cleared from the systemic circulation resulting in accumulation of total IL17A (secukinumab‐IL17A complexes plus free IL‐17A), which reach a plateau at 6 months.^18^ Hence, while the IL‐17 enzyme‐linked immunosorbent assay is suitable to determine total IL‐17A, it does not allow the discrimination between biological active (free IL‐17) and inactive (complexed) IL‐17. Importantly, however, the therapy seemed to decrease the Th17 milieu as evidenced by reduced serum IL‐23 and IL‐22 levels. Moreover, we observed reduced levels of keratinocyte‐derived proinflammatory cytokines, resulting in less severe skin inflammation. While the IL‐17 antagonists may thus reduce the skin Th17 inflammation, it does not affect the production of Th1 cytokines, and it might not improve the intrinsic epidermal barrier defects due to the ABCA12 deficiency.^19^ IL‐profiling in skin biopsies of the patient would have been advantageous but was not performed because deemed too invasive in this context.

## 6 CONCLUSIONS

In this individual trial, secukinumab was effective and well tolerated in the treatment of a child with severe erythrodermic ichthyosis. Clinical efficacy was accompanied by a consistent shift in the cytokine profile in the patient serum.

To our knowledge, this is the first report of the use of secukinumab in a paediatric patient with ABCA12 deficiency‐related severe erythrodermic ARCI. Further studies including more patients and for longer duration are needed to evaluate the use of this promising therapeutic approach in congenital ichthyosis, in terms of sustained efficacy and safety, especially in children and patients with syndromic subtypes.

## CONFLICT OF INTEREST

The authors declare that there are no conflict of interests.

## Supporting information

Supporting Information S1
