# Supplementary material for: Efficacy and safety of secukinumab for the treatment of severe ABCA12 deficiency‐related ichthyosis in a child
Source: Skin Health Dis. 2021 May 3;1(2):e25. doi: 10.1002/ski2.25 (PMC9060064; doi:10.1002/ski2.25)
Supplement: Supplementary file 1 — Supporting Information S1 [file SKI2-1-e25-s001.docx]

**Efficacy and safety of Secukinumab for the treatment of severe ABCA12 deficiency-related ichthyosis in a child**

J. Yogarajah^1,*^ MD, C. Gouveia^1,2,*^ MD, J. Iype^3^ PhD, S. Häfliger^1,2^ MD, A. Schaller^4^ PhD, J.M. Nuoffer^1,3^ MD, M. Fux^3^ PhD, M. Gautschi^1,3^ MD PhD

**Supplementary Online Content**

**Figure S1**

**Case history**

**eMethods.**

**eReferences.**

**Figure S1**


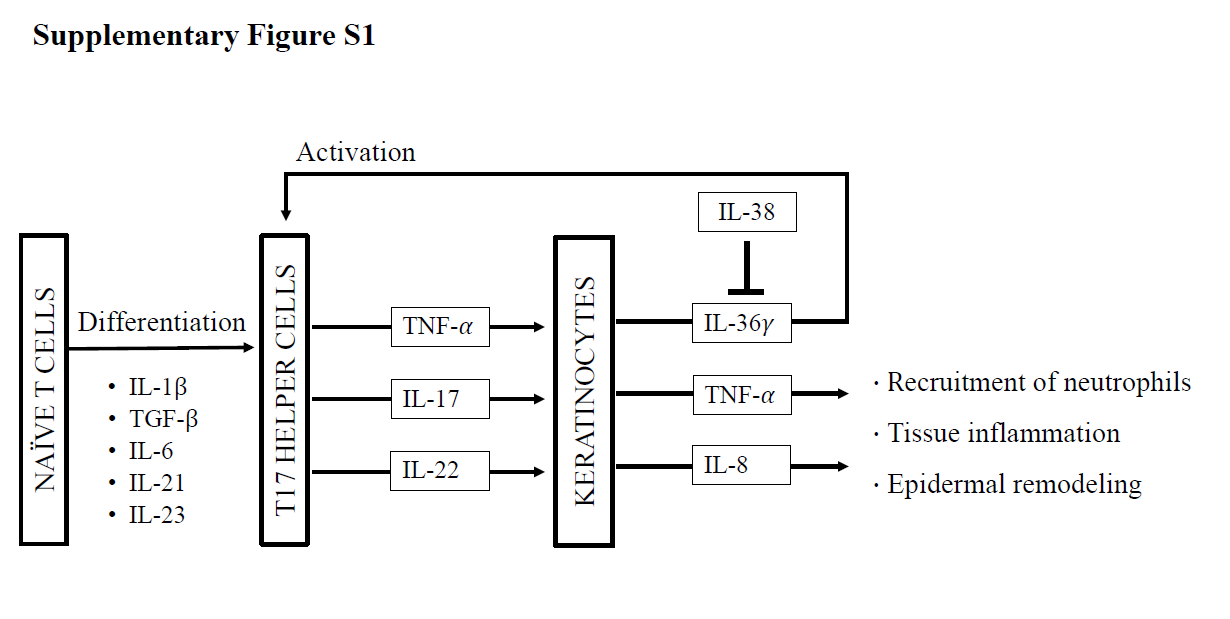


Origin and function of the main cytokines in inflammatory skin disorders. IL-17A from Th17 cells stimulates production of pro-inflammatory mediators such as IL-8 (R1) and IL-36γ (R2) by keratinocytes, promoting recruitment of neutrophils and activation of endothelial cells, respectively. IL, Interleukin; Th cells, T helper cells.

**Case history**

The patient is the second child of non-consanguineous parents. He was born prematurely fully enclosed by a thin collodion membrane (GW 33 1/7, BW 2320g). As the membrane started to shed, generalized ichthyosis with scaling and an increasing erythematous appearance became evident. Trio whole-exome sequencing identified three heterozygous variants in the *ABCA12*-gene: c.1446A>C p.(Glu482Asp), c.2968A>G p.(Lys990Glu) and c.723dupA p.(Val242Serfs*27). In an extensive manual search, no other potential genetic cause of the disorder could be found. The maternal allele harbours the c.723dupA variant, which has not been described as disease-associated, but does not appear in a population database (gnomAD) either (R3). The variant is classified as likely pathogenic according to the ACMG-guidelines (R4). The paternal allele harbours two variants: c.1446A>C, which has already been described as disease associated and the c.2968A>G variant (R5). The latter variant neither is described as disease-associated nor appears in a population database (gnomAD) and is classified as variant of unknown clinical significance according to the ACMG-guidelines.

The severe skin condition greatly impaired the patients' QOL. In addition to the generalized erythema and scaling (including palmoplantar keratoderma) and the intense pruritus, the patient experienced recurrent episodes of a maculopapular rash with worsening of the itching and pain. Due to ichthyosis-related chronic-recurrent mechanical obstructions of the external auditory canals, the patient underwent ear cleanings every four weeks by an otorhinolaryngologist. Palmoplantar keratoderma had impaired the development of body motor skills. Occupational therapy improved gross and fine motor skills. The ichthyosis-related hypohidrosis limited the patient's physical activity. In addition, the striking aspect of the skin condition was also a psychological burden for the patient.

The basic symptomatic therapy consisted of the application of emollients (Lipikar and Excipial), three times daily. On the scalp, a topical compound containing urea-lactic acid was used. He had as well daily (salt) baths with gentle mechanical removal of scales. On top of his underlying condition, the patient suffered from recurrent episodes of maculopapular rash with severe itching from early age onwards. The rash had no apparent triggers and bacterial and fungal swabs had been repeatedly negative. Add-on therapies included cicalfate cream for the areas of eroded skin and topical steroids (mometasone cream) and calcineurin inhibitors (pimecrolimus 1% cream) for the maculopapular rash. Since October 2018, the rash has been persistent mainly on the big flexures (axillae and groins). The patient experienced the itching and burning sensation on the skin as a significant limitation of the QOL.

In view of the additional recurrent exacerbations with the resulting loss in QOL, the therapeutic trial with secukinumab was performed. The basic symptomatic therapy (without topical steroids), which was continued during the trial, was evaluated retrospectively: Prior to therapy start, the basic therapy took up about two hours per day. With secukinumab, the skin had become less scaly, which reduced the time required for the basic therapy by 20 minutes. Towards the end of the 6-month therapy period, daily bathing could sometimes even be skipped and switched to shower instead, which was a very welcomed change and relief for the patient.

**eMethods**

Prior to therapy start, routine laboratory tests were performed, including blood count, inflammatory parameters (C-reactive protein), liver and kidney function. The patient was screened for antibodies against hepatitis B virus, hepatitis C virus and human immunodeficiency virus. QuantiFERON-TB® test and a chest X-ray were performed to exclude tuberculosis. All age-appropriate immunizations were completed, including 13-valent pneumococcal vaccination. The patient was advised not to receive any live vaccinations during the treatment period.

At every follow-up visit, the patient (and his mother) were asked about his current medical condition, potential study drug reactions and adverse events. The safety of secukinumab was assessed by regular laboratory analyses, which included differential full blood count, liver and kidney function, and fecal calprotectin; thereby screening for common and potential adverse effects reported for the study drug, such as neutropenia, increased liver enzymes and development of inflammatory bowel disorder. The general physical examination focused on detecting infections, including candidiasis.

The clinical response to treatment was monitored with standardized pictures of the skin, and assessed using a battery of clinical scorings: Investigator’s Global Assessment (IGA), ranging from 0 (clear) through to 5 (very severe disease). The clinician evaluated the patient’s Clinical Global Impression (CGI) both prior to and in the course of the study drug use. For the Severity-subcomponent (CGI-S), the clinician rated the severity of the disease, using a range from 1 (normal, not ill at all) through to 7 (among the most extremely ill patients). To estimate the Improvement-subcomponent (CGI-I), the clinician rated the extent of improvement in the patient’s condition compared to the baseline visit, on a scale from 1 (very much improved) through to 7 (very much worsened).

The Ichthyosis-Area-Severity-Index (IASI), the modified scoring system of the Psoriasis-Area-Severity-Index, consists of the two subscores Erythema (IASI-E) and Scaling (IASI-S) and ranges between 0 and 48 (R6). The body is divided into four regions (head and neck, trunk, upper limbs, and lower limbs). A body surface-dependent multiplier is allocated to each body area (R6). For each body region, the severity (0 [none] to 4 [very severe]) of erythema or scaling, respectively, with the extent of the area involved (scored 0 [0%] through to 6 [90-100%]) is assessed. The IASI E/S score is considered as a reliable method to monitor the response to treatment (R6).

Pain was evaluated with Wong-Baker Faces (pain rating scale), on a 6-point scale from 0 to 10. Pruritus was assessed with the Itch Man-Scale, on a scale from 0 (comfortable, no itch) to 4 (itches most terribly; impossible to sit still or concentrate). With the Children’s Dermatology Life Quality Index (CDLQI, on a scale from 0 to 30) the patient’s QOL was estimated. To assess the treatment satisfaction, the abbreviated 9-item Treatment Satisfaction Questionnaire for Medication (TSQM-9) was used.

Measurement of cytokines in serum: Sera were aliquoted and stored at -80°C until immunoassays were performed. Additionally, control sera (C1-C6) were collected in compliance with IRB standards from rest material of six age-matched children with unrelated medical conditions including type 1 diabetes mellitus (T1DM, C1-C3), long-term follow up after leukemia (C4), an unknown mild genetic syndrome (C5), Turner mosaic (C6), respectively. The serum samples were thawed at room temperature, centrifuged and filtered prior to use in order to remove any precipitants. The serum levels of cytokines were measured using commercially available direct enzyme-linked immunosorbent assay (ELISA) kits according to the manufacturer’s instructions: IL-1β and TNF-α (851.610.005, 851.570.005 respectively, both Diaclone, Fleming, France), IL-8 (CHC1303, Invitrogen, USA), IL-17A (HS170, Quantikine HS ELISA kit, R&D Systems, UK), IL-22 and IL-23 (D2200, D2300B,respectively, both Quantikine ELISA kit, R&D Systems, UK), , IL-36-γ and IL-38 (DY2320-05, DY9110-05 respectively, R&D Systems, UK). For IL-17A, IL-22 and IL-23, pre-coated microplates were used. For IL-1β, TNF-α, IL-8, IL-36γ, and IL-38 ELISA microplates (Nunc Maxisorb ELISA plates) were coated with the specific capture antibody diluted in phosphate-buffered saline (PBS) overnight at 4ºC. Wells were then washed three times with wash buffer (0.05% Tween 20 in PBS) and subsequently blocked with block buffer (0.5-5% BSA in PBS) for 2-3 hours at room temperature (RT). Serum samples and standards diluted in assay diluent (1% BSA in PBS) were added to the wells. After 2 hours incubation at RT, any excess unbound analyte was removed by washing. Subsequently for IL-17A, IL-36γ, and IL-38, specific biotinylated detection antibody against the analyte was added and incubated for 1-2 hours at RT. Whereas for IL-1β, TNF-α, and IL-8, specific biotinylated detection antibody was incubated simultaneously with samples and standards, respectively, for 2-3 hours at RT. After extensive washing steps, streptavidin-Horseradish peroxidase (HRP) conjugate was incubated for 20-30min at RT. For IL-22 and IL-23 ELISA, specific polyclonal antibody conjugated to HRP was used. After final washing steps, substrate solution (Tetramethylbenzidine) was incubated at RT for 15-30min or until sufficient blue color had developed. Color development was stopped by the addition of 2N H_2_SO_4_ and the optical density (O.D) of each well was determined using a microplate reader (Cytation5, BioTek) at 450nm. The detection range of the ELISAs were 15.6-500pg/ml for IL-1β, 25-800pg/ml for TNF-α, 12.5-800pg/ml for IL-8, 0.234-15pg/ml for IL-17A, 15.6-1000pg/ml for IL-22, 39.1-2500pg/ml for IL-23, 19-1200pg/ml for IL-36-γ, and 31.3-2000pg/ml for IL-38, respectively.

For data analysis data of the standard curves were extrapolated into a linear range (O.D vs concentration). The level of cytokines in the serum samples were subsequently quantified using linear regression analysis, variable slope, four parameters (Graphpad prism). Data are represented as mean± SEM of the replicates. The average of control subjects, C1-6, was included to mark the cut-off for the respective serum cytokine levels, in which C7 was excluded, as the control subject 7 has an autoimmune disorder. The unpaired non-parametric Kruskal-Wallis test with Dunnett`s multiple group comparison test was used to compare between the groups and the results were considered significant if p-values were <0.05.

**Supplementary References (R1-6):**

R1. Nograles KE, Zaba LC, Guttman-Yassky E, et al. Th17 cytokines interleukin (IL)-17 and IL-22 modulate distinct inflammatory and keratinocyte-response pathways. Br J Dermatol. 2008;159(5):1092-102.

R2. Failla CM, Capriotti L, Scarponi C, et al. Interleukin (IL)-17/IL-36 axis participates to the crosstalk between endothelial cells and keratinocytes during inflammatory skin responses. bioRxiv. 2019:767400.

R3. Karczewski KJ, Francioli LC, Tiao G, et al. The mutational constraint spectrum quantified from variation in 141,456 humans. Nature. 2020 May;581(7809):434-443. doi: 10.1038/s41586-020-2308-7. Epub 2020 May 27.

R4. Richards S, Aziz N, Bale S, et al, ACMG Laboratory Quality Assurance Committee. Standards and Guidelines for the Interpretation of Sequence Variants: A Joint Consensus Recommendation of the American College of Medical Genetics and Genomics and the Association for Molecular Pathology. Genet Med. 2015 May;17(5):405-24. doi: 10.1038/gim.2015.30. Epub 2015 Mar 5.

R5. Scott CA, Plagnol V, Nitoiu D, et al. Targeted Sequence Capture and High-Throughput Sequencing in the Molecular Diagnosis of Ichthyosis and Other Skin Diseases. J Invest Dermatol. 2013 Feb;133(2):573-6. doi: 10.1038/jid.2012.332. Epub 2012 Sep 20.

R6. Paller AS, Renert-Yuval Y, Suprun M, et al. An IL-17-dominant immune profile is shared across the major orphan forms of ichthyosis. J Allergy Clin Immunol. 2017;139(1):152-65.
